# Allopurinol Protects against Ischemia/Reperfusion-Induced Injury in Rat Urinary Bladders

**DOI:** 10.1155/2015/906787

**Published:** 2015-09-27

**Authors:** Ju-Hyun Shin, Kwang Sik Chun, Yong-Gil Na, Ki-Hak Song, Seung Il Kim, Jae-Sung Lim, Gun-Hwa Kim

**Affiliations:** ^1^Department of Urology, School of Medicine, Chungnam National University Hospital, Daejeon 301-721, Republic of Korea; ^2^Department of Surgery, School of Medicine, Chungnam National University Hospital, Daejeon 301-721, Republic of Korea; ^3^Division of Bioconvergence Analysis, Korea Basic Science Institute (KBSI), Daejeon 305-333, Republic of Korea; ^4^Department of Functional Genomics, University of Science and Technology (UST), Daejeon 305-806, Republic of Korea

## Abstract

Bladder ischemia-reperfusion (I/R) injury results in the generation of reactive oxygen species (ROS) and markedly elevates the risk of lower urinary tract symptoms (LUTS). Allopurinol is an inhibitor of xanthine oxidase (XO) and thus can serve as an antioxidant that reduces oxidative stress. Here, a rat model was used to assess the ability of allopurinol treatment to ameliorate the deleterious effects of urinary bladder I/R injury. I/R injury reduced the *in vitro* contractile responses of longitudinal bladder strips, elevated XO activity in the plasma and bladder tissue, increased the bladder levels of tumor necrosis factor-*α* (TNF-*α*), c-Jun N-terminal kinase (JNK), and p38 mitogen-activated protein kinase, reduced the bladder levels of extracellular regulated kinase (ERK), and decreased and increased the bladder levels of Bcl-2 and Bax, respectively. I/R injury also elevated lipid peroxidation in the bladder. Allopurinol treatment in the I/R injury was generated significantly ameliorating all I/R-induced changes. Moreover, an *in situ* fluorohistological approach also showed that allopurinol reduces the generation of intracellular superoxides enlarged by I/R injury. Together, the beneficial effects of allopurinol reducing ROS production may be mediated by normalizing the activity of the ERK, JNK, and Bax/Bcl-2 pathways and by controlling TNF-*α* expression.

## 1. Introduction

Lower urinary tract symptoms (LUTS) are common in both men and women, particularly as they age. Bladder ischemia/reperfusion (I/R) is a common factor contributing to age-related structural and functional changes of the bladder. In benign prostate hyperplasia patients, there is a decrease of blood flow (ischemic change) due to elevated intravesical pressure during voiding and/or the increased pressure of bladder wall during urine filling phase. And then this is followed by an increase in blood flow and oxygen tension after micturition (reperfusion phase) [1, 2]. Atherosclerotic obstructive changes distal to the aortic bifurcation in both sexes also can induce a reduction of LUT blood flow, leading to chronic bladder ischemia [3, 4]. I/R [5] during a micturition cycle and chronic ischemic changes due to atherosclerosis can result in the generation of reactive oxygen species (ROS), produce oxidative stress, and contribute to the development of bladder dysfunction and LUTS.

I/R injury is known to trigger the release of inflammatory mediators such as tumor necrosis factor-*α* (TNF-*α*). For example, the myocardium generates local inflammatory cytokines in response to acute I/R that in turn depress cardiac function [5]. Moreover, the cardiopulmonary by-pass procedure elevates TNF-*α* levels [6]. The most important acute urinary retention complication is oxidative stress due to IR injury and bladder inflammatory response that can lead to bladder dysfunction [7, 8].

Allopurinol, xanthine oxidase (XO) inhibitor, is administered orally in hyperuricemic patients to prevent gout. It also has radical scavenging and cardioprotective effects in a myriad of cardiovascular conditions [9]. Recently, presurgical oral allopurinol treatment was shown to reduce the number of ROS-expressing cardiomyocytes when patients with congenital heart disease underwent surgical correction [10]. However, whether allopurinol treatment can prevent ROS formation when the bladder receives an I/R injury remains unclear.

In the present study we aimed to investigate the possible beneficial activities of allopurinol against ischemia and reperfusion on the injured tissue of urinary bladder by fluorohistological and biochemical analysis.

## 2. Materials and Methods

### 2.1. Animals and Experimental Procedures

Male Sprague-Dawley rats weighing 200 ± 30 g were employed in this study. All animal experiments followed a protocol that was approved by the ethics committee on animal research at Chungnam National University, Daejeon, Korea. Animals were fed standard rat chow and had free access to tap water. They were housed individually in separate cages with wood shaving as bedding at standard laboratory conditions (25 ± 1°C, 55 ± 5% humidity and a 12 h alternating light-dark cycle). The 45 animals in the study were divided into three groups: the saline-pretreated sham-operated group (Sham + S), the saline-pretreated ischemia-reperfusion group (I/R + S), and the allopurinol-pretreated ischemia-reperfusion group (I/R + Allo). The rats were anesthetized by intramuscular injection of xylazine (Rompun, 10 mg/kg) and ketamine (Imalgene, 100 mg/kg). For allopurinol treatment, allopurinol in powder form (Sigma-Aldrich, St. Louis, MO) was dissolved in saline, and 2 M NaOH was added to generate a final pH of approximately 10.5 [11, 12]. The final solution was injected intraperitoneally twice a week for 2 weeks at a concentration of 50 mg/kg of body weight. This allopurinol dose was selected because it was found previously to be safe for the liver in rats [12]. The saline-pretreated groups were injected intraperitoneally twice a week for 2 weeks with a pH 10.5 saline solution. The saline volume was the same as that used for allopurinol.

Two weeks after starting the injections, the rats were placed on a servocontrolled surgical table to reduce heat loss during the surgery. An ischemic insult was generated by clamping the bilateral vesical arteries with atraumatic vascular clips for 2 h under the surgical microscope [13]. Reperfusion was then achieved by removing the clamps. The incisions were closed by using continuous size 4-0 vicryl sutures for peritoneal and muscle closure and size 5-0 silk sutures for skin closure. The sham operation involved the same technique and exposure except that the arteries were not clamped. Thereafter, the rats were injected twice in the following week with saline or 50 mg/kg of allopurinol. At the end of week after the operation, all rats were anesthetized, and blood was collected from both internal iliac veins while the rats were alive. Fresh blood samples from internal iliac veins were used for assessing XO activity. Thereafter, the bladder was removed through an abdominal incision. Bladder removal was performed by separating the bladder dome and body from the bladder base at the level of the ureteral orifices. Bladder tissue was weighed and taken for biochemical measurements and immediately stored at −70°C. Longitudinal strips were then isolated from the remaining bladder tissue and immediately placed in organ baths filled with oxygenated Krebs solution.

### 2.2. Contractile Responses of Bladder Strips

The organ baths, each of which had one longitudinal strip, contained 50 mL of Krebs-Henseleit solution (99.01 mM NaCl, 4.69 mM KCl, 1.89 mM CaCl_2_, 1.2 mM MgSO_4_, 1.03 mM KH_2_PO_4_, 25 mM NaHCO_3_, and 11.1 mM glucose) that was aerated with a mixture of 95% oxygen and 5% carbon dioxide and maintained at 37°C. An initial resting tension of 1 g was applied to the bladder strips for 60 min, and the contractile responses were recorded in an isometric fashion with a force-displacement transducer. The contractile responses to 10^−8^–10^−3^ mol/L carbachol (CCh) were cumulatively obtained and expressed as force per 100 mg of bladder muscle.

### 2.3. XO Activity in Plasma and Bladder Tissue

XO activity in plasma, bladder tissue, or XO standards was measured by using the Amplex Red Xanthine/Xanthine Oxidase Assay Kit (Molecular Probes, Eugene, OR) according to the manufacturer's instructions. This kit measures XO activity on the basis of colorimetric change in Amplex Red at 560 nm in the presence of hypoxanthine. The background absorbance is subtracted. XO catalyzes the oxidation of hypoxanthine or xanthine to superoxide, which spontaneously degrades to hydrogen peroxide. In the presence of horseradish peroxidase, the hydrogen peroxide reacts with Amplex Red to generate the red oxidation product, which is called resorufin. The lower limit of detection in this assay is 0.1 mU/mL.

### 2.4. TNF-*α* Levels in Bladder Tissue

The bladder tissue was homogenized in RIPA (Radio-Immunoprecipitation Assay) lysis buffer containing 1 mmol/L PMSF, and a supernatant was obtained by centrifugation (18 000 ×g at 4°C for 15 min). The TNF-*α* levels in the supernatant were quantified by using a rat TNF-*α* sandwich enzyme-linked immunoassay kit (R&D Systems, Minneapolis, MN, USA).

### 2.5. Western Blotting of Bladder Tissue

After bladder tissues were homogenized in RIPA buffer, whole cell homogenates were centrifuged at 13,000 ×g, 4°C for 20 min and the supernatants were collected. Each supernatant was loaded onto acrylamide gels and separated by SDS-PAGE. The proteins in the gel were then transferred onto a nitrocellulose membrane, after which the membranes were stained with Ponceau to check for uniform protein loading. After blocking the membranes, they were incubated overnight at 4°C with the following antibodies: JNK (#3708), Phospho-JNK (#9251), p38 (#9212), Phospho-p38 (#9211, Cell Signaling Technology, MA, USA), xanthine oxidase (SC-22006), ERK1 (SC-94), Phospho-ERK1 (SC-7383), Bax (SC-493), Bcl-2 (SC-7382), and GAPDH (SC-25778, Santa Cruz Biotechnology, CA, USA).

### 2.6. Malondialdehyde (MDA) Levels in Bladder Tissue

The extent of lipid peroxidation was measured by determination of the amounts of MDA. MDA amounts were assayed with a thiobarbituric acid reactive substances (TBARS) kit (ZeptoMetrix), which is based on a spectroscopic method. MDA levels were performed according to standard protocol as previously described [14].

### 2.7. Dihydroethidium Stainings for* In Situ* Detections of Superoxides

To evaluate production of intracellular superoxides using* in situ* dihydroethidium fluorescence (DHE, Invitrogen, Eugene, OR, USA), paraffin-embedded tissues (5 mm) were incubated with 10 mmol/L DHE in phosphate-buffered saline in a dark, humidified container at 37°C for 30 min. The generation of superoxide radicals in the tissues was demonstrated by visualization and quantification of red fluorescence intensities using an LSM 710 confocal microscope (Carl Zeiss, Jena, Germany). The generation of superoxide radicals by tissue was demonstrated by a red fluorescent signal, and the density of the images was reported as arbitrary fluorescence units per square millimeter of field [15].

### 2.8. Statistical Analysis

The data were expressed as mean ± standard error of the mean. The three groups were compared in terms of contractile responses by repeated measures ANOVA and in terms of other variables by the Mann–Whitney *U* test. SPSS version 18.0 for Windows was used to evaluate the data. Differences were considered statistically significant if the null hypothesis could be rejected with >95% confidence (*P* < 0.05).

## 3. Results

**Figure 1:**
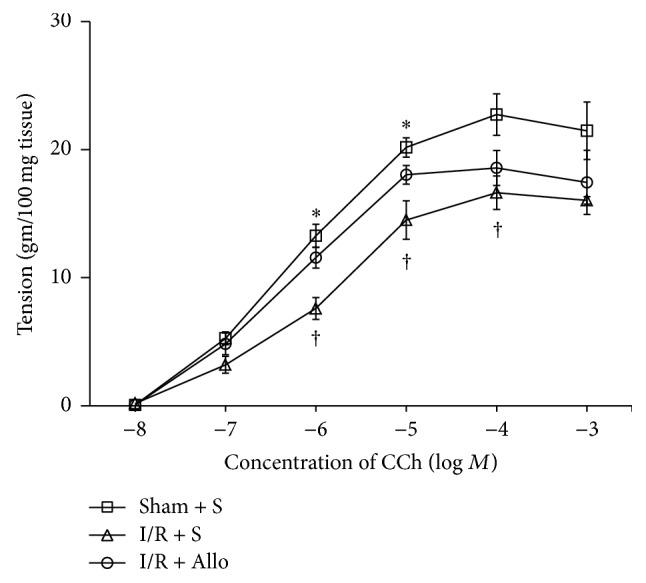
Concentration-contraction curves obtained by cumulative addition of carbachol (CCh) to bladder strips. Each point represents the mean ± SEM of 15 bladder strips. ∗ indicates that I/R + Allo group is significantly different from I/R + S group (*P* < 0.05). † indicates that I/R + S group is significantly different from sham-operated groups (*P* < 0.01). Values are expressed as gram tension per 100 mg wet tissue. Sham-operated plus saline-pretreated group (Sham + S), ischemia-reperfusion and saline-pretreated group (I/R + S), and ischemia-reperfusion and allopurinol-pretreated group (I/R + Allo).

To determine the effects of allopurinol on bladder contractile dysfunctions induced by I/R injury, longitudinal bladder strips from the Sham + S, I/R + S, and I/R + allopurinol (Allo) rat groups were placed in individual organ baths and stimulated with 10^−8^ to 10^−3^ M carbachol (CCh; muscarinic receptor stimulation). Indeed, the bladder strips of the I/R + S group exhibited significantly smaller contractile responses to CCh than the Sham + S group (Figure 1). Interestingly, however, allopurinol treatment of the I/R rats (I/R + Allo) significantly restored the contractile responses to CCh.

**Figure 2:**
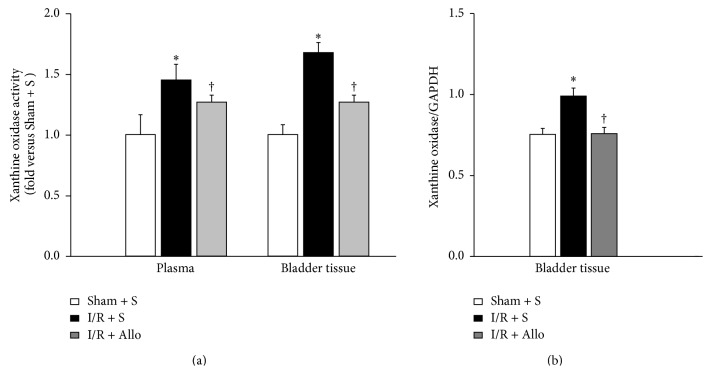
(a) Results of xanthine oxidase (XO) in plasma and bladder tissue. Values are expressed as mU/mg protein. In each of the experiments, XO activity obtained with Sham + S group was normalized as 1.0. (b) Protein levels of XO in bladder tissue. Expression level of xanthine oxidase was normalized to GAPDH. Bars indicated mean ± SEM of 15 rats. ∗ indicates that I/R + S group is significantly different from sham-operated groups (*P* < 0.01). † indicates that I/R + Allo group is significantly different from I/R + S group (*P* < 0.05).

Xanthine oxidase (XO) is major source of ROS, especially superoxides (O^2−^), in mammalian tissue [16]. To determine whether bladder I/R injury induces XO-derived ROS and allopurinol regulates ROS generation in I/R bladder, the XO activity in the plasma and bladder tissue was measured (Figure 2(a)). Indeed, the I/R + S group had 1.45 ± 0.13-fold higher plasma XO activity levels than the Sham + S group (*P* = 0.01). Intriguingly, this I/R-induced increase was slightly reduced by allopurinol treatment (1.27 ± 0.06-fold, *P* = 0.048). This pattern was also observed in the bladder tissues: the I/R + S group had 1.68 ± 0.10-fold higher bladder tissue XO activity than the Sham + S group (*P* = 0.008), but allopurinol treatment reduced this increase (1.27 ± 0.06-fold, *P* = 0.037). As shown in Figure 2(b), the mean levels of XO protein in bladder tissue showed a marked increase following the I/R + S group compared with the Sham + S group. Interestingly, XO proteins were significantly decreased in the I/R + Allo group compared with the I/R + S group.

**Figure 3:**
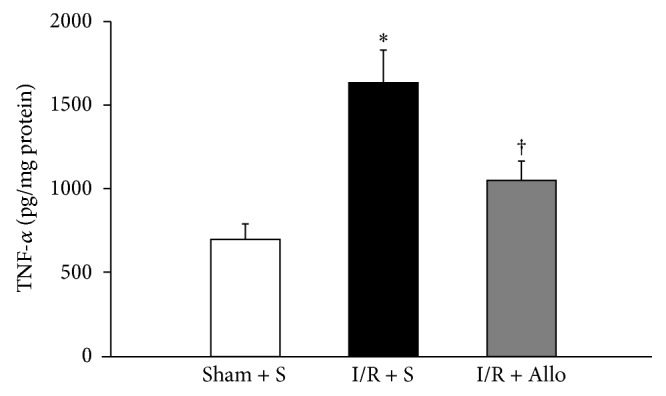
TNF-*α* protein level in the rat urinary bladder. Each bar represents the mean ± SEM. Values are expressed as pg/mg protein. ∗ indicates that TNF-*α* level in I/R + S group is significantly different from Sham + S group (*P* < 0.01). † indicates that I/R + Allo group is significantly different from I/R + S group (*P* < 0.05).

Next, we examined whether allopurinol treatments can control TNF-*α* expression, an indicator of inflammatory response, in bladders that have been subjected to I/R injury. The mean levels of TNF-*α* showed a marked increase in the I/R + S group compared with the S + S group (Figure 3, lanes 1 and 2; Sham + S: 701.8 ± 93.2; I/R + S: 1638.8 ± 195.5 pg/mg protein; *P* = 0.001). This increase of I/R + S group in inflammatory change was abolished by allopurinol administration (Figure 3, lane 3; I/R + Allo: 1054.6 ± 116.0 pg/mg protein; *P* = 0.030).

**Figure 4:**
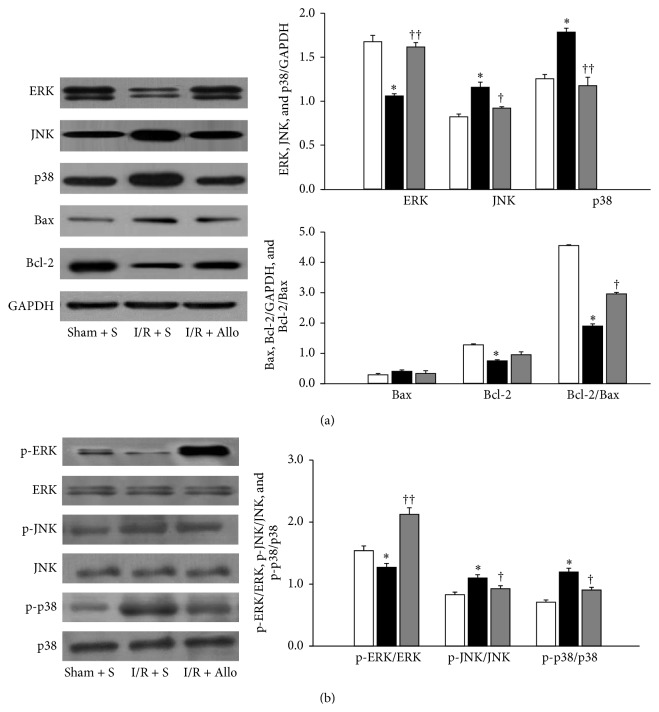
Effect of allopurinol on MAPKs and apoptosis pathway. (a) Western blot analysis of ERK, JNK1, p38, Bax, and Bcl-2. Expression levels of each protein were normalized to GAPDH. (b) Western blot analysis of ERK, Phospho-ERK, JNK, Phospho-JNK, p38, and Phospho-p38. ^∗^ *P* < 0.05, I/R + S group compared with the sham-operated group. ^†^ *P* < 0.05, ^††^ *P* < 0.01, I/R + S group versus I/R + Allo. Data show mean ± SEM of *n* = 15 replicates.

We continued to investigate whether this drug regulates MAPKs (ERK, JNK, and p38) and apoptotic pathway in bladder I/R injury. Compared to the Sham + S group, the I/R + S group had significantly higher JNK and p38 protein levels and significantly lower ERK levels, in the bladder. Interestingly, allopurinol treatment significantly reduced the I/R-induced increase in JNK and p38 protein levels while simultaneously elevating ERK protein levels. The I/R + S group also exhibited significantly less antiapoptotic Bcl-2 expression and significantly more proapoptotic Bax expression than the Sham + S group. As a result, the I/R + S group had a significantly lower Bcl-2/Bax ratio than the Sham + S group. Allopurinol treatment significantly ameliorated these changes in Bcl-2 and Bax expression and the Bcl-2/Bax ratio (Figure 4(a)). Moreover, allopurinol treatment consistently reduced the I/R-induced increase in Phospho-JNK and Phospho-p38 protein levels while simultaneously elevating Phospho-ERK protein levels (Figure 4(b)).

**Figure 5:**
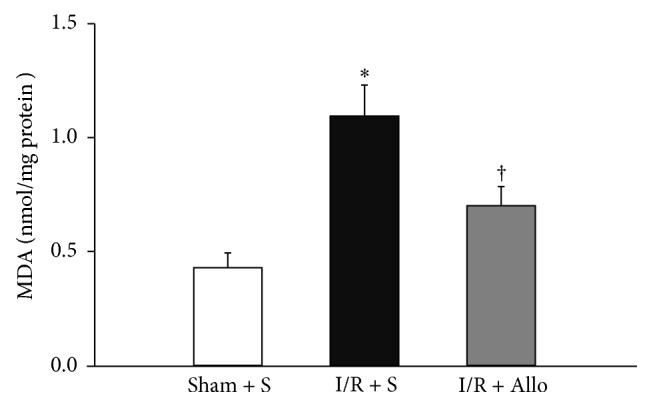
Malondialdehyde (MDA) levels in bladder tissue. ∗ indicates that I/R + S group is significantly different from sham-operated groups (*P* < 0.01). † indicates that I/R + Allo group is significantly different from I/R + S groups (*P* < 0.05). Bars indicate mean ± SEM. Values are expressed as nmol/mg protein.

Recently, we showed that bladder I/R injury leads to the formation of lipid peroxidation [14]. Considering these results, the above finding, which indicated that allopurinol had antioxidant effects, prompted us to determine whether allopurinol treatments can also regulate the levels of lipid peroxidation. As shown in Figure 5, the mean levels of malondialdehyde (MDA), a marker for lipid peroxidation levels, showed a marked increase following the I/R + S group compared with the Sham + S group in bladder (Sham + S: 0.43 ± 0.06 nmol/mg protein; I/R + S: 1.09 ± 0.13 nmol/mg protein; *P* = 0.001). Lipid peroxidation in I/R + S group was largely inhibited by treatment with allopurinol (0.70 ± 0.07 nmol/mg protein; *P* = 0.027).

**Figure 6:**
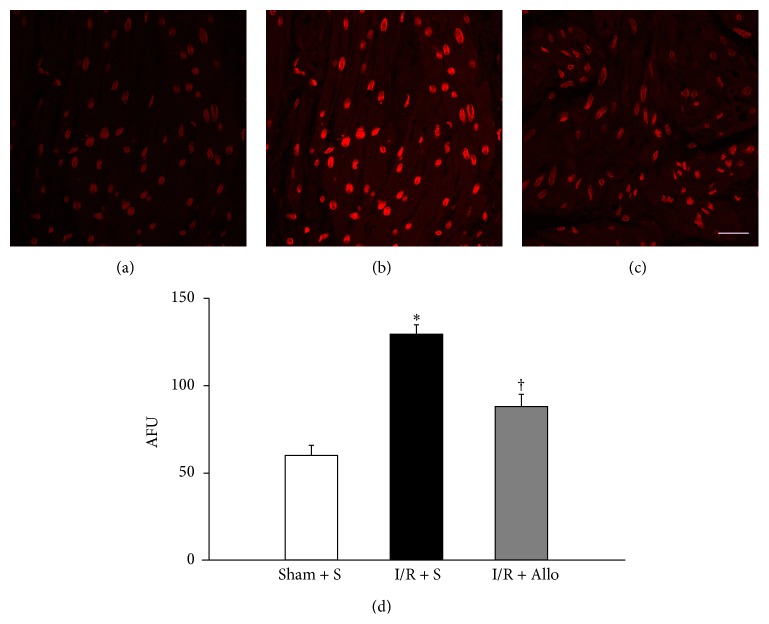
Detection of* in situ* superoxide in bladder tissue by DHE staining. I/R + S group showed significantly increased signal intensities compared with Sham + S group. Compared with I/R + S group, the DHE fluorescence intensity in the bladder of I/R + Allo group was significantly decreased. Images were acquired at identical settings and are representative of similar results obtained from three independent experiments. (a) Sham + S; (b) I/R + S; (c) I/R + Allo. The scale bar indicates 50 mm (bottom). (d) Intensity values are expressed as arbitrary fluorescence units. ∗ indicates that I/R + S group is significantly different from sham-operated groups (*P* < 0.05). † indicates that I/R + Allo group is significantly different from I/R + S groups (*P* < 0.05). Bars indicate mean ± SEM.

Consistent with this biochemical result, semiquantitative analyses of fluorescence using DHE staining (Figure 6), which measure* in situ* production of superoxides, revealed significantly increased signal intensities in the I/R + S group compared with the Sham + S group (Figures 6(a), 6(c), and 6(d); S + S: 59.8 ± 5.8 arbitrary fluorescence units; I/R + S: 129.2 ± 5.4 arbitrary fluorescence units; *P* = 0.012). This generation of superoxides in I/R + S group was largely inhibited by treatment with allopurinol (Figures 6(c) and 6(d); I/R + S: 87.8 ± 7.0 arbitrary fluorescence units; *P* = 0.029).

## 4. Discussion 

Allopurinol is known to protect against cardiovascular disease by reducing the formation of superoxide anion of XO and by directly scavenging free radicals and chelating non-protein-bound iron [17]. The ability of allopurinol to reduce I/R damage in cardiology and in pediatric and adult cardiothoracic surgery has long been established [17, 18]. In the present study, we showed a new application of allopurinol that acts as a pharmacological compound that can reverse the low contractile bladder responses and can prevent oxidative stress following I/R injury.

Since ROS can activate inflammation, several studies examined whether allopurinol treatment can inhibit the inflammatory pathways by decreasing ROS production. Indeed, in the liver, allopurinol pretreatment before I/R damage reduces the liver levels of TNF-*α*, an inflammatory cytokine [19]. Cardiocytes also produce TNF-*α* in response to ischemia, and TNF-*α* plays a critical role in the myocardial dysfunction that arises after acute injury [20]. Inhibiting XO via allopurinol treatment improves endothelium-dependent dilation and reduces the superoxide production of isolated coronary arterioles after I/R damage [21]. Our present study first showed that allopurinol treatment suppressed TNF-*α* protein production induced by I/R damage of the bladder, which further illustrates the protective effect of allopurinol.

I/R injury leads to the formation of ROS, which can destroy nucleic acids, proteins, and carbohydrates as well as the lipid layer on the cell membrane. The latter destruction produces lipid radical peroxyl and lipid peroxide, which in turn induces the formation of MDA that is one of the end products of the decomposition of lipid peroxidation products and is often used as a biomarker of oxidative damage [22]. Thus, allopurinol treatment is expected to reduce the destruction of lipid in the cell membrane. Indeed, bladder I/R injury elevated MDA levels in the bladder, and this effect was ameliorated by allopurinol treatment. This result is consistent with studies that show that ROS scavenging prevents the lipid peroxidation process in the reperfusion phase.

I/R-induced tissue injury has been related to activation of MAPKs pathway [23], but bladder tissue is unclear. In the present study, we showed that bladder I/R injury associates with the activation of p38 and JNK (the so-called death pathway), but not ERK. By contrast, allopurinol treatment significantly increased the expression of ERK and reduced p38 and JNK levels. Many studies suggest that members of the Bcl-2 family, especially Bcl-2 and Bax, are key regulators of physiological and pathological apoptosis via the activation of caspase cascade. Consistent with our data, the ratio of Bcl-2/Bax protein is important to determine myocardial survival or death after I/R [24, 25]. In addition, it has been known that activation of the ERK pathway inhibits the conformational change in Bax and therefore prevents apoptosis [26]. Together, these results suggest that the greater activity of the prosurvival kinase ERK compared to p38 and JNK may favor cell survival after I/R insult.

In conclusion, the present study showed that XO-mediated ROS formation may play an important role in the pathogenesis of bladder I/R injury and that allopurinol protects against apoptotic state by modulating the ERK, JNK, p38, Bax, and Bcl-2 pathways. Moreover, allopurinol inhibits the production of TNF-*α*, thereby reducing inflammation. Therefore, allopurinol may be useful in strategies aiming to protect the bladder from I/R injury.
